# Pimonidazole-alkyne conjugate for sensitive detection of hypoxia by Cu-catalyzed click reaction

**DOI:** 10.1007/s44211-024-00520-y

**Published:** 2024-03-13

**Authors:** Iori Tamura, Daichi M. Sakamoto, Bo Yi, Yutaro Saito, Naoki Yamada, Yoichi Takakusagi, Shinsuke Sando

**Affiliations:** 1 Department of Chemistry and Biotechnology, Graduate School of Engineering, The University of Tokyo, 7-3-1 Hongo, Bunkyo-ku, Tokyo, 113-8656 Japan; 2 Quantum Hyperpolarized MRI Research Team, Institute for Quantum Life Science, National Institutes for Quantum Science and Technology, 4-9-1 Anagawa, Inage, Chiba-City, 263-8555 Japan; 3 Institute for Quantum Medical Science, National Institutes for Quantum Science and Technology, 4-9-1 Anagawa, Inage, Chiba-City, 263-8555 Japan; 4 Department of Bioengineering, Graduate School of Engineering, The University of Tokyo, 7-3-1 Hongo, Bunkyo-ku, Tokyo, 113-8656 Japan; 5 Present Address: Department of Physiology, National Defense Medical College, 3-2 Namiki, Tokorozawa, Saitama 359-8513 Japan

**Keywords:** Hypoxia probe, Pimonidazole, Click chemistry, Fluorescence imaging, Tumor hypoxia

## Abstract

Hypoxia is involved in various diseases, such as cancers. Pimonidazole has often been used as the gold-standard marker to visualize hypoxic regions. Pimonidazole labels hypoxic regions by forming a covalent bond with a neighboring protein under hypoxic conditions in the body, which is detected by immunohistochemistry performed on tissue sections. To date, some pimonidazole-fluorophore conjugates have been reported as fluorescent probes for hypoxia imaging that do not require immunostaining. They are superior to pimonidazole because immunostaining can produce high background signals. However, large fluorophores in the conjugates may alter the original biodistribution and reactivity. Here, we report a new hypoxia marker, Pimo-yne, as a pimonidazole-alkyne conjugate. Pimo-yne has a similar hypoxia detection capability as pimonidazole because the alkyne tag is small and can be detected by Cu-catalyzed click reaction with azide-tagged fluorescent dyes. We successfully visualized hypoxic regions in tumor tissue sections using Pimo-yne with reduced background signals. The detected regions overlapped well with those detected by pimonidazole immunohistochemistry. To further reduce the background, we employed a turn-on azide-tagged fluorescent dye.

**Graphical abstract:**

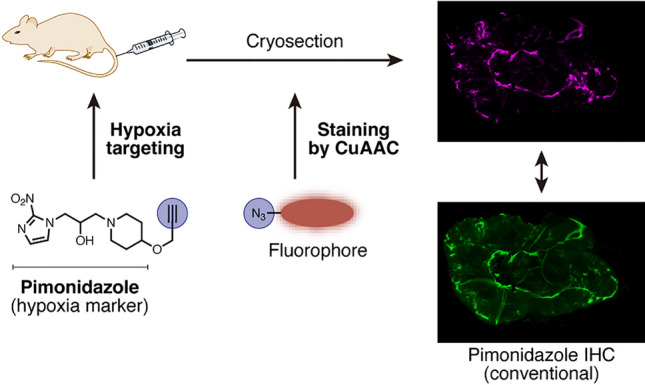

**Supplementary Information:**

The online version contains supplementary material available at 10.1007/s44211-024-00520-y.

## Introduction

**Fig. 1:**
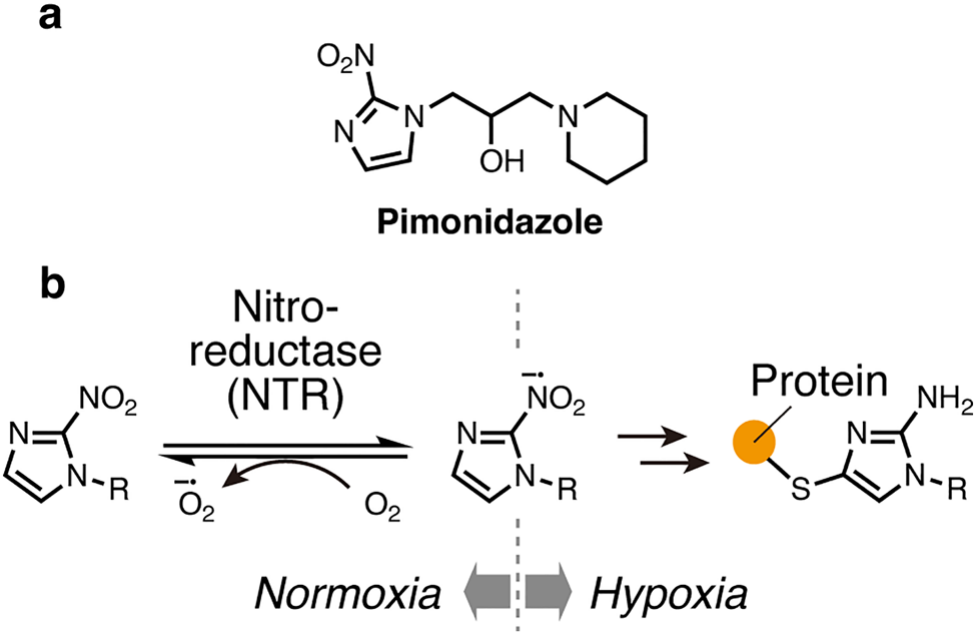
Chemical structure and mechanism of hypoxia detection of pimonidazole, a common hypoxia marker. **a** Chemical structure of pimonidazole. **b** Proposed mechanism of hypoxia-dependent labeling of proteins by nitroimidazole compounds

Hypoxia plays an important role in the pathology of various diseases, such as cancers and neurodegenerative diseases [1, 2]. For example, intratumoral hypoxia is involved in angiogenesis, metastasis, and radioresistance [3, 4]. Hypoxia imaging techniques with molecular markers have contributed to elucidating mechanisms of hypoxia-related phenomena. Notably, pimonidazole (Fig. 1a) [5] has been widely used as the gold-standard hypoxia marker. Pimonidazole contains a 2-nitroimidazole group, which is reduced by nitroreductases (NTRs) and converted to an electrophilic species under hypoxia. Subsequently, the electrophilic species reacts with a nucleophilic residue of a neighboring protein to form a covalent bond, resulting in the chemical labeling of hypoxic regions (Fig. 1b) [6]. Following the administration of pimonidazole in vivo, the labeled hypoxic regions can be visualized on tissue sections by immunohistochemistry (IHC) for pimonidazole-protein adducts. However, IHC has concerns regarding background signals due to the non-specific binding or false-positive signals from the antibody cross-reactivity.

**Fig. 2:**
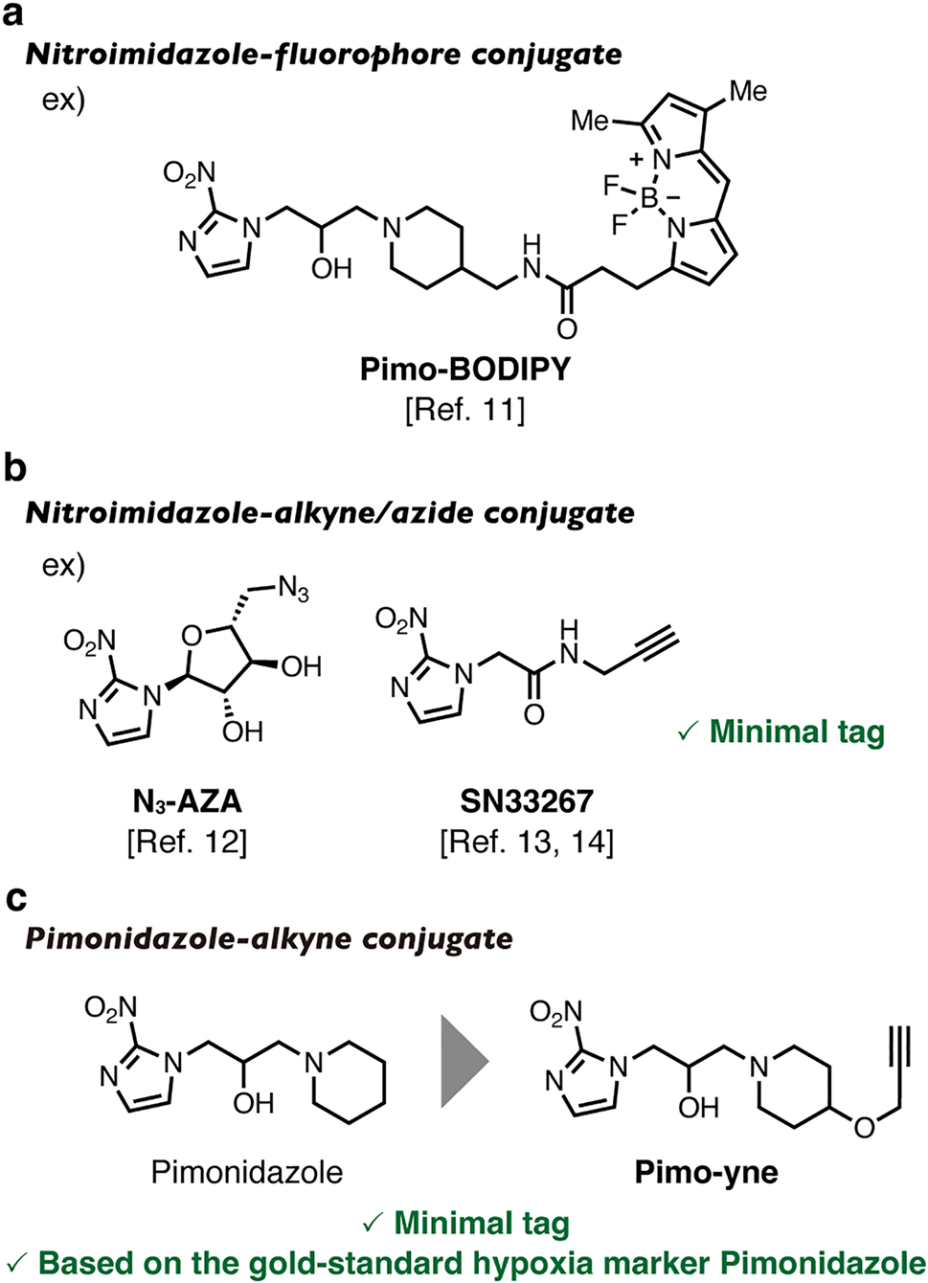
Previous examples of hypoxia probes and the molecular probe Pimo-yne developed in this work. **a** A previous example of nitroimidazole-fluorophore conjugates. **b** Previous examples of nitroimidazole-alkyne/azide conjugates. **c** A pimonidazole-alkyne conjugate, Pimo-yne, developed in this work

In this context, some nitroimidazole-fluorophore conjugates have been reported as superior hypoxia probes (Fig. 2a) [7–11]. For example, a nitroimidazole-cyanine 7 (Cy7) conjugate [8] and a nitroimidazole-indocyanine green (ICG) conjugate [10] were developed and used for in vivo imaging and tissue section imaging of hypoxia. Most recently, we reported a pimonidazole-BODIPY conjugate and a pimonidazole-Me-Si-Rhodol conjugate [11]. These pimonidazole-fluorophore conjugates covalently bind to neighboring proteins under hypoxia conditions to label hypoxic regions with fluorophores, thus serving as hypoxia markers without IHC. Despite this advantage, their large fluorophores could adversely affect the biodistribution and hypoxia responsiveness of the probes.

To address these problems, we adopted click chemistry to the hypoxia labeling process. An alkyne and an azide function as minimal click handles and selectively react with each other through bio-orthogonal Cu-catalyzed azide-alkyne cycloaddition (CuAAC), which is the most common click reaction. Following hypoxia labeling by an alkyne- or azide-tagged hypoxia-responsive probe, the labeled hypoxic regions can be detected by CuAAC reaction with a fluorescent dye harboring the corresponding reaction moiety such as azide and alkyne. This sequential process can realize tissue hypoxia imaging with little or no alterations to the pharmacokinetic properties of the hypoxia-responsive probe since the alkyne and azide groups are very small. To date, some nitroimidazole-alkyne/azide conjugates have been reported (Fig. 2b) [12–15]. Rashed et al*.* reported azidoazomycin arabinoside (N_3_-AZA) as an azide derivative of ^18^F-fluoroazomycin arabinoside (^18^F-FAZA), a tracer of positron emission tomography (PET) for hypoxia [12]. Intratumor hypoxia on tissue sections was successfully detected by CuAAC reaction between N_3_-AZA and an alkyne-tagged fluorescent dye. Tercel et al*.* reported SN33267 as a simple nitroimidazole-alkyne conjugate, and Hou successfully detected intratumor hypoxia on tissue sections by hypoxia labeling with SN33267 and CuAAC reaction with an azide-tagged fluorescent dye [13, 14]. These probes possess great potential as alternatives to pimonidazole and nitroimidazole-fluorophore conjugates for the reasons mentioned above. However, due to differences in the chemical structures of these probes from pimonidazole, there are potential concerns that these probes show different behaviors on hypoxia labeling from the gold-standard marker pimonidazole.

**Fig. 3:**
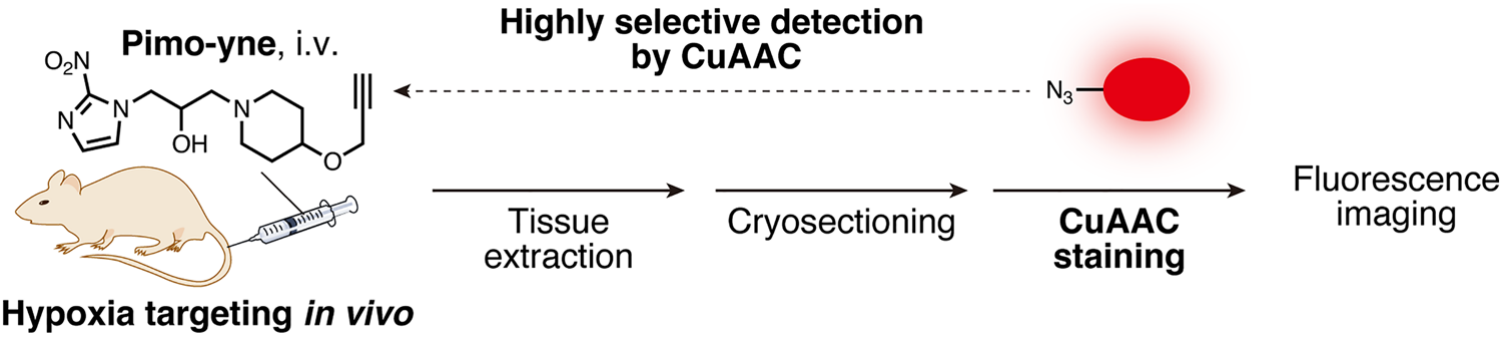
Schematic illustration of hypoxia imaging by Pimo-yne, coupled with CuAAC staining

Here, we developed an alkyne-tagged pimonidazole, Pimo-yne, as a new clickable hypoxia probe (Fig. 2c). Since the alkyne tag was one of the minimal tags, Pimo-yne was expected to behave similarly to pimonidazole. Pimo-yne was synthesized and examined the ability to label hypoxic cells, which resulted in high hypoxia selectivity. Furthermore, as shown in Fig. 3, in vivo hypoxia imaging was evaluated by a selective CuAAC reaction with azide-tagged fluorescent dyes on tissue sections from the Pimo-yne-administered mice. In the imaging, comparable staining regions to pimonidazole were observed with lower background signals. For further reduction of the background signals, we tested a turn-on azide fluorescent dye, CalFluor 647 (CF647) azide [16].

## Experimental

### General

Unless otherwise noted, all materials used in this study were purchased from commercial suppliers and used without further purification. Unless otherwise noted, work-up and purification procedures were performed with reagent-grade solvents under air. Analytical thin-layer chromatography (TLC) was performed using E. Merk silica gel 60 F_254_ precoated plates (0.25 mm). The developed chromatogram was analyzed with a UV lamp (254 nm) and ethanolic ninhydrin. Flash column chromatography was performed with an Isolera Spektra instrument equipped with a Biotage® Sfär Silica HC D 10 g or Sfär Silica HC D 50 g cartridge. Preparative high performance liquid chromatography (HPLC) was performed on a Prominence HPLC system (Shimadzu) with a 5C18-AR-II column (#38150-41, Nacalai tesque). High-resolution mass spectrometry (HRMS) was measured using a Bruker micrOTOF II (ESI). Nuclear magnetic resonance (NMR) spectra were recorded on a JEOL ECS400 (^1^H 400 MHz, ^13^C 100 MHz) spectrometer. Chemical shifts for ^1^H NMR are expressed in parts per million (ppm) relative to CHCl_3_ (δ 7.26 ppm) in CDCl_3_, HOD (δ 4.79 ppm) in D_2_O. Chemical shifts for ^13^C NMR are expressed in ppm relative to CDCl_3_ (δ 77.19 ppm) in CDCl_3_. Data are reported as follows: chemical shift, multiplicity (s = singlet, d = doublet, t = triplet, m = multiplet), coupling constant (Hz), and integration.

### Synthetic procedures

*Synthesis of tert-butyl 4-(prop-2-yn-1-yloxy)piperidine-1-carboxylate (2).* Compound **2** was synthesized according to the previous literature with minor modifications [17].

*Synthesis of 4-(prop-2-yn-1-yloxy)piperidine hydrochloride (3).* Compound **3** was synthesized according to the previous literature with minor modifications [18].

*Synthesis of 2-nitro-1-(oxiran-2-ylmethyl)-1H-imidazole (5).* Compound **5** was synthesized according to the previous literature with minor modifications [9].

*Synthesis of Pimo-yne.* 2-Nitro-1-(oxiran-2-ylmethyl)-1*H*-imidazole (**5**, 560 mg, 3.31 mmol, 1.0 equiv.) and 4-(prop-2-yn-1-yloxy)piperidine hydrochloride (**3**, 640 mg, 3.64 mmol, 1.1 equiv.) were suspended in anhydrous ethanol (20 mL). The mixture was refluxed with stirring for 1 h. The solution was concentrated under reduced pressure, and the remaining solid was purified with flash silica-gel chromatography (MeOH/EtOAc = 5:95–40:60) to yield Pimo-yne (256 mg, 830 μmol, 25%) as a pale yellow solid. ^1^H NMR (400 MHz, CDCl_3_): δ 7.28 (d, *J* = 1.0 Hz, 1H), 7.13 (d, *J* = 1.0 Hz, 1H), 4.68 (dd, *J* = 2.5, 14.0 Hz, 1H), 4.27–4.22 (m, 1H), 4.17 (d, *J* = 2.4 Hz, 2H), 4.05–4.00 (m, 1H), 3.62–3.56 (m, 1H), 2.88–2.83 (m, 1H), 2.67–2.62 (m, 1H), 2.50–2.40 (m, 3H), 2.26–2.15 (m, 2H), 1.93–1.86 (m, 2H), 1.68–1.54 (m, 2H); ^13^C NMR (100 MHz, CDCl_3_): δ 145.0, 128.2, 127.6, 80.2, 74.2, 73.4, 65.9, 60.4, 55.3, 53.5, 51.7, 50.5, 31.2, 31.1; HRMS (ESI): Calculated for C_14_H_21_N_4_O_4_ [M + H]^+^ 309.1557, found 309.1545.

*Synthesis of Cy5-PEG*_*4*_*-azide.* Cyanine 5 (Cy5)-COOH trifluoroacetate (**6**) was synthesized according to the previous literature with minor modifications [19–21]. Cy5-COOH trifluoroacetate (**6**, 78.9 mg, 102 μmol, 1.0 equiv.), amino-PEG_4_-azide (34.2 mg, 130 μmol, 1.3 equiv.), 1-ethyl-3-(3-dimethylaminopropyl)carbodiimide (EDCI) hydrochloride (27.9 mg, 146 μmol, 1.4 equiv.), 1-hydroxybenzotriazole (HOBt) hydrate (25.2 mg, 165 μmol, 1.6 equiv.), and *N*,*N*-diisopropylethylamine (DIPEA) (82.6 μL, 474 μmol, 4.6 equiv.) were dissolved in anhydrous *N*,*N*-dimethylformamide (DMF) (*ca.* 1.2 mL). The reaction mixture was stirred at room temperature (r.t.) for 23 h. The solvents were concentrated under reduced pressure. The residue was purified with preparative HPLC (25% to 40% MeCN/H_2_O with 0.1% trifluoroacetic acid) to yield Cy5-PEG_4_-azide trifluoroacetate (43.8 mg, 43.1 μmol, 42%) as a deep blue solid. ^1^H NMR (400 MHz, D_2_O): δ 8.06–7.98 (m, 2H), 7.85–7.79 (m, 4H), 7.31 (dd, *J* = 8, 14 Hz, 2H), 6.53 (t, *J* = 12 Hz, 1H), 6.21 (dd, *J* = 14, 25 Hz, 2H), 4.11–4.02 (m, 4H), 3.64–3.58 (m, 14H), 3.51 (t, *J* = 5 Hz, 2H), 3.42–3.39 (m, 2H), 3.28 (t, *J* = 5 Hz, 2H), 2.22 (t, *J* = 7 Hz, 2H), 1.82–1.75 (m, 2H), 1.68–1.57 (m, 14H), 1.36–1.29 (m, 5H); HRMS (ESI): Calculated for C_43_H_59_N_6_O_11_S_2_ [M–H]^–^ 899.3689, found 899.3652.

*Synthesis of pimonidazole hydrochloride.* Pimonidazole hydrochloride was synthesized according to the previous literature with minor modifications [22, 23].

### Reaction monitoring of CuAAC between Cy5-PEG_4_-azide and alkynes by ultra high performance liquid chromatography (UHPLC)

To a 1.5 mL tube, 420 μL of Dulbecco's phosphate buffer saline (–) (DPBS(–)), 34.5 μL of dimethyl sulfoxide (DMSO), 10 μL of 100 mM 2-(4-((bis((1-(*tert*-butyl)-1*H*-1,2,3-triazol-4-yl)methyl)amino)methyl)-1*H*-1,2,3-triazol-1-yl)acetic acid (BTTAA) in DMSO, 5 μL of 100 mM CuSO_4_ in H_2_O, 0.5 μL of 10 mM Cy5-PEG_4_-azide in DMSO, and 5 μL of 10 mM alkyne (Pimo-yne or propargyl alcohol) in DMSO were added in this order with vortexing after each addition. Then, 25 μL of 100 mM sodium ascorbate (NaAsc) in DPBS(–) was added followed by vortexing to start the reaction. The final concentrations of the reagents were 10 μM Cy5-PEG_4_-azide, 100 μM alkyne, 1 mM CuSO_4_, 2 mM BTTAA, 5 mM NaAsc, 10% v/v DMSO, in a total volume of 500 μL. At each time point (2, 5, 10, 30, and 60 min after starting the reaction), 50 μL of 0.5 M ethylenediaminetetraacetic acid (EDTA) (pH 8.0) was added followed by vortexing to quench the reaction. For t = 0 min, EDTA was added before the addition of the NaAsc solution. The solution was analyzed by UHPLC on LC-2040C 3D Plus (Shimadzu) with a Shim-pack Velox C18 column (#227-32007-02, Shimadzu).

### Measurement of fluorescence spectra during CuAAC between CF647 azide and alkynes

To a quartz cell, 2655 μL of DPBS(–), 231 μL of DMSO, 60 μL of 100 mM BTTAA in DMSO, 30 μL of 100 mM CuSO_4_ in H_2_O, 3 μL of 1 mM CF647 azide in DMSO, and 6 μL of 10 mM alkyne (Pimo-yne or propargyl alcohol) in DMSO were added in this order and stirred by a magnetic stirrer. The fluorescence spectra were measured at this time as t = 0 min. Immediately after 15 μL of 1 M NaAsc in DPBS(–) was added, the fluorescence spectra were measured every 3 min for 60 min. The final concentrations of the reagents were 1 μM CF647 azide, 20 μM alkyne, 1 mM CuSO_4_, 2 mM BTTAA, 5 mM NaAsc, 10% v/v DMSO, in a total volume of 3 mL. The solution was kept at 25 °C and stirred continuously during the reaction. The fluorescence spectra were measured by an RF-6000 (Shimadzu).

### Cell culture

SCCVII and C26 cells were cultured in Roswell Park Memorial Institute (RPMI)-1640 medium supplemented with 10% fetal bovine serum and 1% antibiotic–antimycotic (#09366-44, Nacalai Tesque, Kyoto, Japan) in 5% CO_2_ in an incubator (MCO-18AC, Panasonic Healthcare) at 37 °C.

### Confocal laser scanning microscope (CLSM) imaging

SCCVII cells were seeded at a density of 2.4 × 10^4^ cells/well on an 8-well chamber (#5232-008, IWAKI) and allowed to adhere overnight. For hypoxia labeling, the cells were incubated in RPMI-1640 containing 1% v/v DMSO and 100 μM Pimo-yne or pimonidazole hydrochloride in 1% O_2_ (hypoxia) or *ca.* 20% O_2_ (normoxia) and 5% CO_2_ at 37 °C for 5 h. For hypoxia, the cells were incubated in a multi-gas incubator (MCO-5M, SANYO). The cells were washed with DPBS(–) twice and further incubated in probe-free medium in *ca.* 20% O_2_ and 5% CO_2_ at 37 °C for 2 h. The cells were washed with DPBS(–) twice and fixed with 4% w/v paraformaldehyde (PFA) in 0.1 M phosphate buffer (PB) at r.t. for 10 min. Washed with DPBS(–) twice, the cells were permeabilized with 0.2% w/v Triton X-100 in DPBS(–) at r.t. for 10 min × 3, and then washed with DPBS(–) twice. For CuAAC staining as shown in Fig. 5b, the cells were treated with a CuAAC reaction solution (25 μM Cy5-PEG_4_-azide or CF647 azide, 1 mM CuSO_4_, 2 mM BTTAA, 5 mM NaAsc, 10% v/v DMSO in DPBS(–)) in the dark at r.t. for 1 h, and then washed with 0.2% w/v Triton X-100 in DPBS(–) 5 min × 3 and DPBS(–) once. For immunocytochemistry as shown in Fig. S2, the cells were blocked with Protein Block Serum-Free (#X0909, Dako) at r.t. for 30 min and treated with fluorescein isothiocyanate (FITC)-conjugated anti-pimonidazole mouse monoclonal antibody (1:200 in blocking solution, #HP MAb-1, Hypoxyprobe) in the dark at 4 °C overnight, and then washed with DPBS(–) 5 min × 3. After each staining, the cells were treated with 1:1000 Hoechst 33342 (#H342, DOJINDO) diluted in DPBS(–) for nuclear staining. Finally, the cells were imaged with CLSM (TCS SP8, Leica). The acquired images were processed by Fiji [24].

### In-gel fluorescence imaging

SCCVII cells (3.0 × 10^4^ cells) were seeded on a 35 mm dish (#150460, Thermo Scientific), allowed to adhere overnight, and incubated for probe labeling as described above. After 2 h incubation in a probe-free medium, the cells were washed with DPBS(–) twice and lysed by adding cell lysis buffer (#9803, Cell Signaling Technology) and scraping. The lysate was centrifuged, and the supernatant was collected. To 86 μL of lysate, a pre-mixed cocktail of 5.5 μL of DMSO, 2 μL of 100 mM BTTAA in DMSO, and 2.5 μL of 1 mM Cy5-PEG_4_-azide or CF647 azide in DMSO was added. After adding 3 μL of 100 mM CuSO_4_ in H_2_O and 1 μL of 500 mM NaAsc in H_2_O, the lysate was shaken in the dark at 700 rpm and 25 °C for 1 h. After that, 2 μL of 0.5 M EDTA (pH 8.0) was added to quench the reaction, and proteins in the lysate were precipitated by acetone, and the pellet was washed with acetone to remove the CuAAC reagents. After resolubilization by cell lysis buffer, sodium dodecyl sulfate-polyacrylamide gel electrophoresis (SDS-PAGE) was performed with SuperSep Ace Mini, 10–20%, 17well (FUJIFILM Wako). The in-gel fluorescence was imaged by a gel imager (iBright FL1500, Invitrogen), followed by Coomassie Brilliant Blue (CBB) staining.

### Flow cytometry

SCCVII cells were seeded at a density of 2.0 × 10^5^ cells/well on a 12-well plate (#2-8588-02, VIOLAMO), allowed to adhere overnight, and incubated for probe labeling as described above except for the probe concentration being 1 μM. After 2 h incubation in a probe-free medium, the cells were washed with DPBS(–) twice and dissociated with TrypLE Express (Gibco). The cell suspension was added RPMI, centrifuged twice, and fixed in 2% w/v PFA in PB in the dark at r.t. for 15 min. After adding 1% w/v bovine serum albumin in DPBS(–) and centrifuging, the cells were permeabilized in 0.3% w/v Tween-20 in DPBS(–) in the dark on ice for 30 min. DPBS(–) was added to the cell suspension and the suspension was centrifuged. To the cell pellet was added a CuAAC reaction solution (25 μM Cy5-PEG_4_-azide or CF647 azide, 1 mM CuSO_4_, 2 mM BTTAA, 5 mM NaAsc, 10% v/v DMSO in DPBS(–)). The mixture was shaken in the dark at 300 rpm and 25 °C for 1 h. The cells were washed by centrifuging in DPBS(–) and 50% v/v EtOH/DPBS(–), resuspended by DPBS(–), and analyzed by a flow cytometer (Guava easyCyte, Merck Millipore).

### Tumor-bearing mouse model and tissue section imaging

BALB/c mice (female, 8-week-old, purchased from CLEA Japan) were prepared. C26 cells (*ca.* 1 × 10^6^ cells) were suspended in serum-free RPMI-1640 and subcutaneously inoculated to both hindlegs of the mice. After 10 days, Pimo-yne and pimonidazole hydrochloride were dissolved in the vehicle (5% v/v DMSO, 45% v/v polyethylene glycol (PEG)-400, 50% v/v DPBS(–)) and intravenously injected into the mice at 30 mg/kg each. After 2 h, the mice were anesthetized by isoflurane and perfused by sterile heparinized DPBS(–) and 4% PFA in 0.1 M PB. The tumors were extracted and fixed in 4% PFA in 0.1 M PB at 4 °C overnight. After DPBS(–) wash, the tumor tissues were immersed in sucrose solution, embedded in optimal cutting temperature (OCT) compound (#4583, Sakura Finetek Japan), and frozen on dry ice. The samples used in the following experiment were randomly chosen from the two tumors obtained from each leg of the mice. The tissue sections at a 10 μm thickness were prepared with a cryostat (CM1950, Leica) and permeabilized with 0.2% w/v Triton X-100 in DPBS(–) for 10 min. After DPBS(–) wash, the sections were treated with a CuAAC reaction solution (25 μM Cy5-PEG_4_-azide or CF647 azide, 1 mM CuSO_4_, 2 mM BTTAA, 5 mM NaAsc, 10% v/v DMSO in DPBS(–)) in the dark at r.t. for 1 h and washed with 0.2% w/v Triton X-100 in DPBS(–) three times. Then, the sections were washed with DPBS(–) three times and blocked with Protein Block Serum-Free in the dark at r.t. for 30 min. After the blocking solution was removed, the sections were incubated with anti-pimonidazole rabbit polyclonal antibody (1:100 in blocking solution, #Pab2627, Hypoxyprobe) in the dark at 4 °C overnight. (NOTE: Pimo-yne was not detected by the anti-pimonidazole antibody (data not shown)). The sections were washed with DPBS-T (0.05% v/v Tween-20 in DPBS(–)) three times and incubated with Alexa Fluor 488 (AF488)-conjugated anti-rabbit antibody (1:100 in blocking solution, #4412S, Cell Signaling Technology). The sections were washed with DPBS-T three times and mounted with DAPI fluoromount-G (#0100-20, SouthernBiotech). The sections were imaged by a fluorescence microscope (BZ-X800, Keyence) with Plan Apochromat 4X (BZ-PA04) or Plan Apochromat 10X (BZ-PA10). The acquired images were processed using Fiji [24].

## Results and discussion

### Synthesis of a pimonidazole derivative with an alkyne tag

**Scheme 1:**
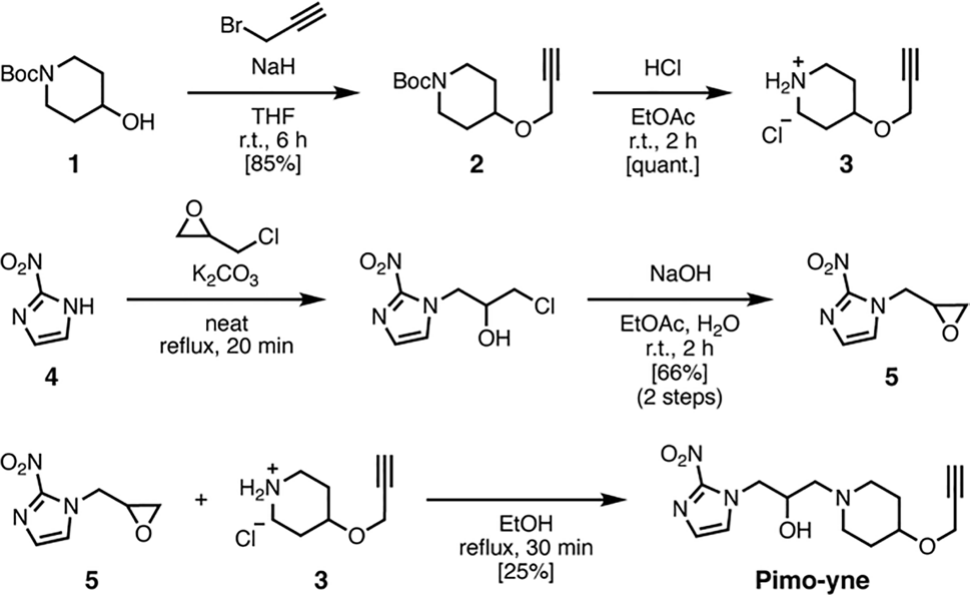
Synthesis of Pimo-yne

Pimo-yne was designed as a hypoxia probe candidate with an alkyne tag on the pimonidazole skeleton. Pimo-yne has a propargyl group on the 4-position of the piperidine ring via ether oxygen. This compound was synthesized according to Scheme 1. Piperidine hydrochloride **3** was prepared from *N*-*tert*-butoxycarbonyl (Boc)-protected piperidin-4-ol (**1**) through Williamson ether synthesis with propargyl bromide, followed by removal of the Boc group. Epoxide intermediate **5** was prepared from 2-nitroimidazole (**4**) through a ring-opening reaction of epichlorohydrin under neat conditions and treatment with NaOH. Pimo-yne was synthesized by a coupling of piperidine hydrochloride **3** and epoxide intermediate **5** in 17% overall yield over three steps from **4**.

### Evaluation of CuAAC reaction between Pimo-yne and azide-tagged fluorescent dyes in vitro

**Fig. 4:**
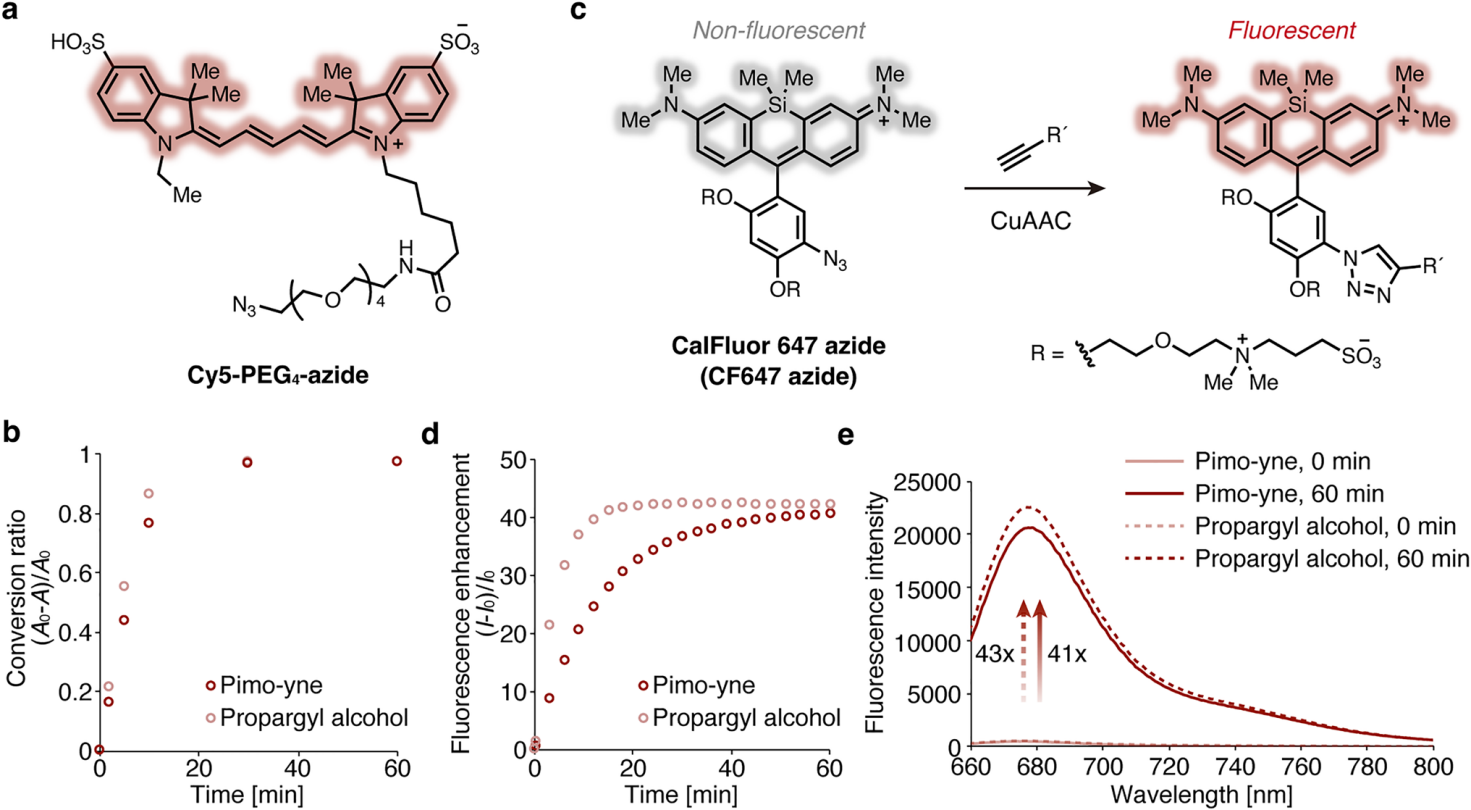
Reaction of Pimo-yne with azide-tagged fluorescent dyes. **a** Chemical structure of Cy5-PEG_4_-azide. **b** Time course of CuAAC reaction between Cy5-PEG_4_-azide and Pimo-yne or propargyl alcohol analyzed by UHPLC (absorbance at 640 nm). *A*: Peak area of Cy5-PEG_4_-azide at time t. *A*_0_: Peak area of Cy5-PEG_4_-azide at t = 0 min. **c** CuAAC reaction of CalFluor 647 azide (CF647 azide) with alkynes. **d** Time course of CuAAC reaction between CF647 azide and Pimo-yne or propargyl alcohol analyzed by fluorescence change using fluorescence spectrometer (excitation: 650 nm, emission: 678.5 nm (Pimo-yne) or 676.5 nm (propargyl alcohol)). *I*: Fluorescence intensity of CF647 at time t. *I*_0_: Fluorescence intensity of CF647 at t = 0 min. **e** Fluorescence spectra of CF647 azide before and after a 60 min CuAAC reaction with Pimo-yne or propargyl alcohol (excitation: 650 nm)

The CuAAC reaction between Pimo-yne and azide-tagged fluorescent dyes was tested in vitro. As an azide-tagged fluorescent dye, Cy5-PEG_4_-azide (Fig. 4a) was designed to employ a Sulfo-Cy5 fluorophore and a PEG linker, which are both hydrophilic to avoid non-specific binding to tissues. In addition, Cy5 has a longer excitation wavelength, which minimizes the influence of the tissue autofluorescence on imaging. Cy5-PEG_4_-azide was synthesized through condensation of Cy5-COOH with amino-PEG_4_-azide in 42% yield (Scheme S1). To a DPBS(–) solution of 10 μM Cy5-PEG_4_-azide, 1 mM CuSO_4_, 2 mM BTTAA (as a Cu(I)-stabilizing ligand), and 100 μM Pimo-yne or propargyl alcohol (as a simple model substrate with alkyne), 5 mM NaAsc (as a reductant for Cu(II)) was added to start the CuAAC reaction. The reaction solution was quenched by the addition of excess EDTA and analyzed by UHPLC. As a result, Pimo-yne exhibited comparable reactivity to propargyl alcohol and almost complete conversion within 30 min under our experimental conditions (Figs. 4b, S1).

Next, we focused on CF647 azide, which is a turn-on azide-tagged fluorescent dye (Fig. 4c, left). The fluorescence of CF647 is quenched via Photo-induced electron Transfer (PeT), whereas the fluorescence is enhanced when the azide group is converted to the triazole group through CuAAC reaction, leading to reducing the PeT effect (Fig. 4c, right) [16]. CF647 azide was reported to undergo approximately 46-fold fluorescence enhancement by CuAAC reaction with an alkyne. A series of CalFluors [16] including CF647 were expected to contribute to more sensitive imaging with lower background signals because they have turn-on fluorescence properties and hydrophilic zwitterion linkers. Among them, CF647 has the longest excitation wavelength, which was promising for suppressing autofluorescence.

Prior to utilizing CF647 azide for hypoxia imaging, we assessed its reactivity with Pimo-yne and propargyl alcohol. The reaction was performed by adding 5 mM NaAsc solution to a DPBS(–) solution of 1 μM CF647 azide, 1 mM CuSO_4_, 2 mM BTTAA, and 20 μM Pimo-yne or propargyl alcohol. The fluorescence spectra were measured every 3 minutes for 60 minutes. The reaction was almost complete after 60 minutes in both cases (Fig. 4d). Compared to propargyl alcohol, Pimo-yne displayed somewhat slower reaction. This is probably due to the steric repulsion from pimonidazole moiety. Nevertheless, the reaction successfully proceeded with almost the same fluorescence enhancement ratio as propargyl alcohol, which is comparable to the reported value in the case of another model alkyne [16]. The fluorescence spectra of CF647 after the CuAAC reaction with both alkynes were matched well (Fig. 4e). This indicates that the Pimo-yne structure did not significantly affect the photophysical properties of CF647 after the reaction.

### Cellular assay of Pimo-yne

**Fig. 5:**
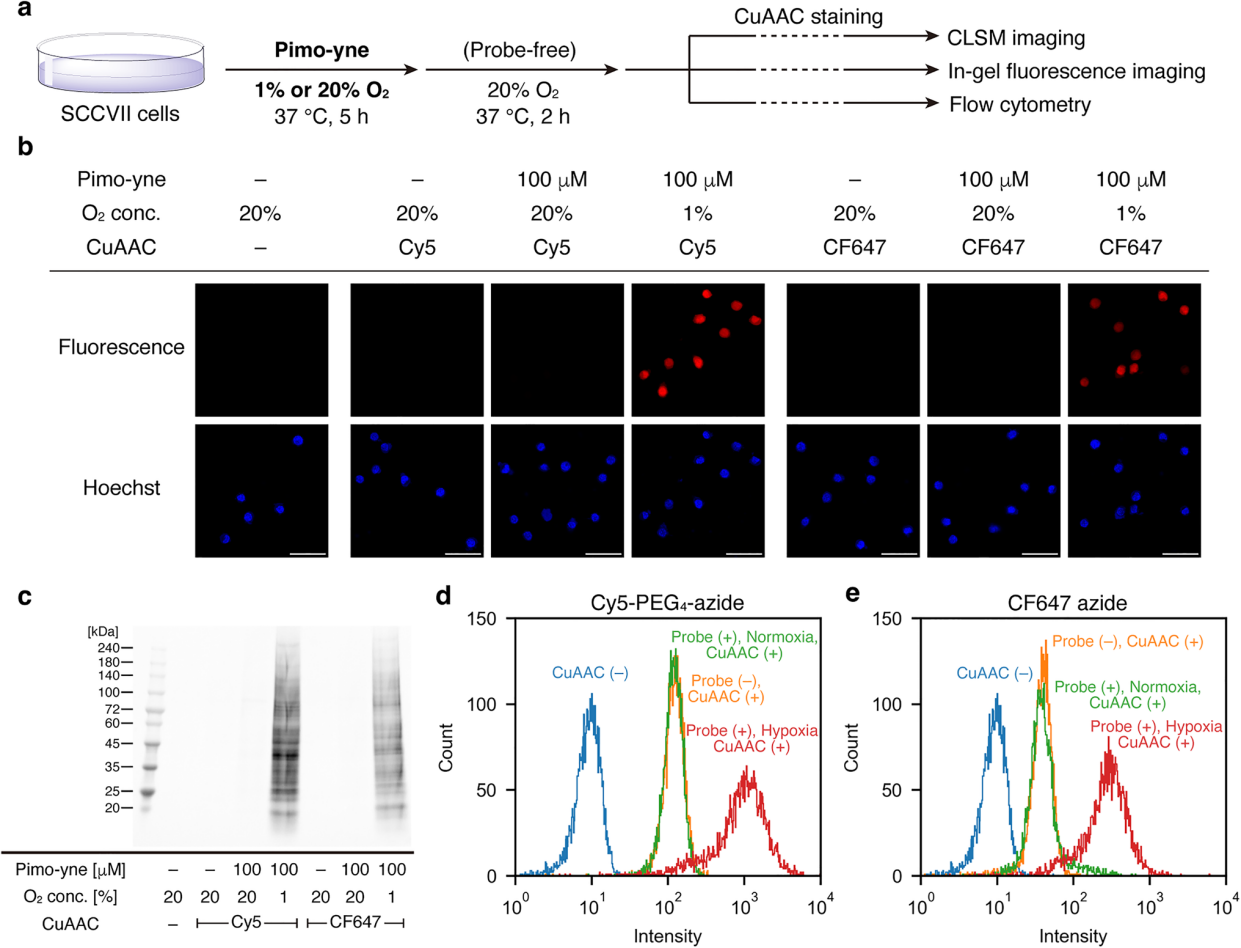
Cellular assay on hypoxia detectability of Pimo-yne. **a** Schematic illustration of the cellular assay. **b** CLSM imaging of cells treated with Pimo-yne under hypoxia or normoxia, followed by CuAAC labeling with azide-fluorophores. Scale = 50 μm. **c** In-gel fluorescence imaging of SDS-PAGE for lysates of cells treated with Pimo-yne under hypoxia or normoxia, followed by CuAAC labeling with azide-fluorophores. **d,e** Flow cytometry analysis of cells treated with Pimo-yne under hypoxia or normoxia, followed by CuAAC labeling with **d** Cy5-PEG_4_-azide and **e** CF647 azide

To evaluate the ability of Pimo-yne to label hypoxic cells, cellular assays were conducted (Fig. 5a). SCCVII cells were incubated under hypoxia (1% O_2_) or normoxia (*ca.* 20% O_2_) for 5 h in the presence of 100 μM Pimo-yne, followed by 2-h incubation under normoxia in the absence of Pimo-yne to remove unbound compounds from the cells. After fixation, permeabilization, and CuAAC staining with Cy5-PEG_4_-azide or CF647 azide, the cells were imaged by CLSM. The results showed that the cells incubated under hypoxia were selectively stained with Pimo-yne and either dye (Fig. 5b). The generation of a hypoxic environment was validated by the conventional immunocytochemistry for pimonidazole (Fig. S2). Both Cy5-PEG_4_-azide and CF647 azide, on their own, exhibited little staining on cells, indicating they did not cause non-specific binding to cellular proteins.

To confirm that the hypoxia-selective signals were derived from the covalent labeling of Pimo-yne to proteins, in-gel fluorescence imaging was conducted. After incubation with Pimo-yne under the same conditions as CLSM imaging, the cells were lysed and CuAAC staining was conducted with Cy5-PEG_4_-azide or CF647 azide in the lysate. Unreacted reagents were removed through acetone precipitation and resolubilization of the proteins, followed by SDS-PAGE. The results of in-gel fluorescence imaging proved that the proteins were stained with a high hypoxia selectivity (Figs. 5c, S3). This indicates that Pimo-yne labeled diverse cellular proteins in a hypoxia-dependent manner as expected.

To quantitatively evaluate the hypoxia selective labeling of Pimo-yne, flow cytometry analysis was conducted. SCCVII cells were incubated under hypoxia or normoxia for 5 h in the presence of 1 μM Pimo-yne, followed by 2-h incubation under normoxia in the absence of Pimo-yne. After detachment, fixation, permeabilization, and CuAAC staining with Cy5-PEG_4_-azide or CF647 azide, the cells were analyzed by flow cytometry. The results demonstrated a high hypoxia selectivity of both dyes (Figs. 5d, e). Cy5-PEG_4_-azide and CF647 azide resulted in comparable hypoxia/normoxia ratios of the median values of 8.4 and 7.5, respectively ([Probe (+), Hypoxia, CuAAC (+)]/[Probe (+), Normoxia, CuAAC (+)]). Despite the low concentration (1 μM) of Pimo-yne, the results exhibited selectivity enough to distinguish between hypoxic and normoxic cells. This could be attributed to the suppression of background signals derived from non-specific binding to cellular proteins of the dyes because Cy5-PEG_4_-azide and CF647 azide have a hydrophilic fluorophore (in the case of Cy5) and linker. In addition, the turn-on property of CF647 was also expected to contribute to background reduction. However, the signal-to-background ratio of the CuAAC staining ([Probe (+), Hypoxia, CuAAC (+)]/[Probe (–), Normoxia, CuAAC (+)]) were 8.2 and 7.3 for Cy5-PEG_4_-azide or CF647 azide, respectively. Thus, turn-on detection by CF647 had no significant effect on further background reduction under these experimental conditions.

### Evaluation of Pimo-yne as a pimonidazole alternative using a tumor-bearing mouse model

**Fig. 6:**
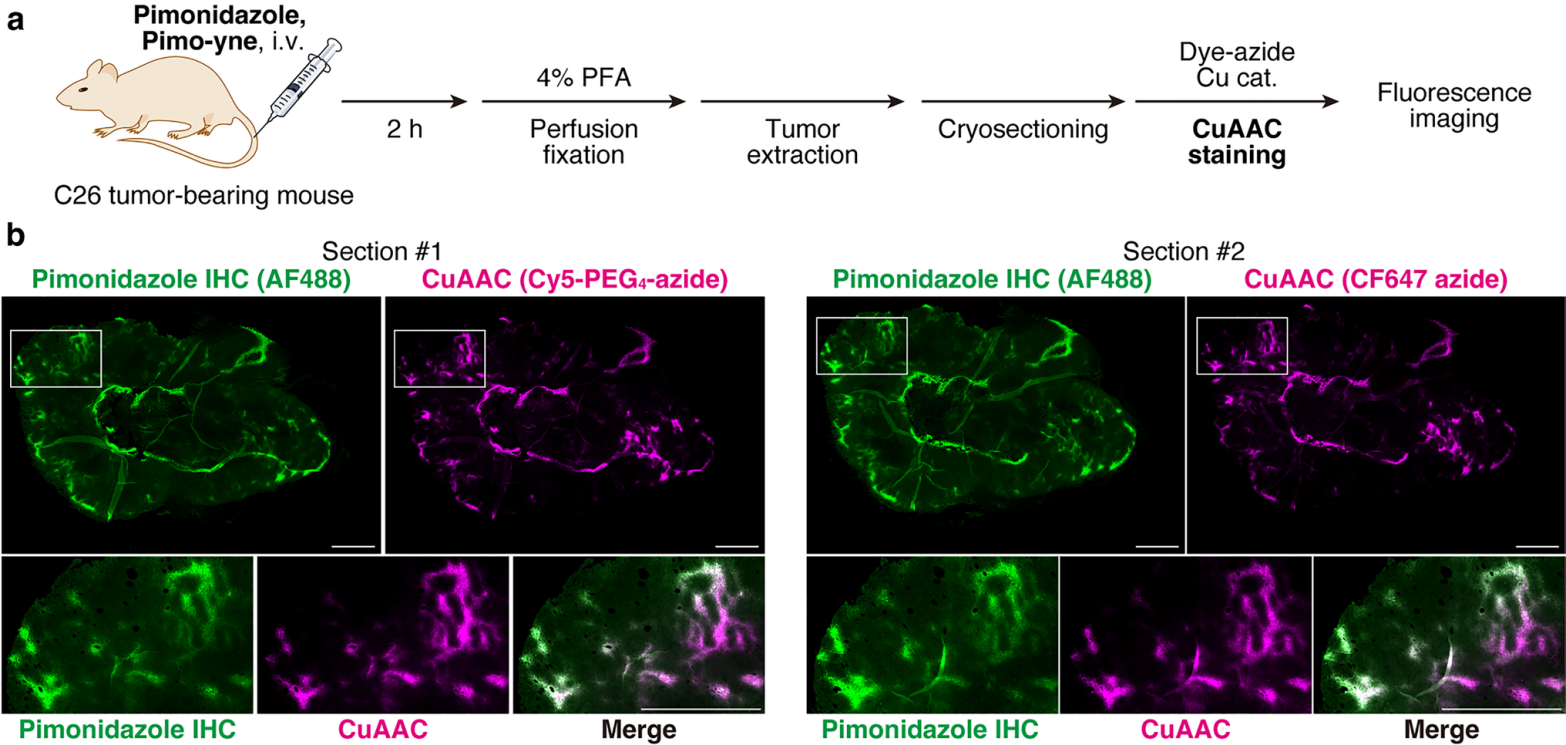
C26 tumor section imaging using Pimo-yne coupled with CuAAC staining. **a** Schematic illustration of the experiment. **b** Multi-color imaging of Pimo-yne (CuAAC with Cy5-PEG_4_-azide or CF647 azide, magenta) and pimonidazole (IHC with anti-pimonidazole primary antibody and AF488-conjugated secondary antibody, green). Lower images show zoomed-in views of the white boxes in upper images. Magnification: × 10. Scale = 1 mm

To evaluate the ability of Pimo-yne to detect hypoxic regions in vivo, tumor tissue section imaging of hypoxia was conducted using a tumor-bearing mouse model (Fig. 6a). C26 tumor-bearing mice were intravenously administered Pimo-yne and pimonidazole (30 mg/kg each) or vehicle, and transcardially perfused with DPBS(–) and 4% PFA. Frozen tissue sections were prepared from the extracted tumors and stained by CuAAC reaction for Pimo-yne and IHC for pimonidazole. In Fig. 6b, the pimonidazole-positive regions stained by IHC are shown in green, while the Pimo-yne-positive regions stained by CuAAC reaction are shown in magenta. The signals derived from Pimo-yne and pimonidazole matched well with either dye. These results demonstrate that Pimo-yne can label hypoxic regions similarly to pimonidazole. On the other hand, IHC for pimonidazole caused background signals, whereas CuAAC staining for Pimo-yne exhibited subtle background signals (Fig. S4). The results of the vehicle control samples indicate that the high background signals of the IHC were most likely due to non-specific binding or cross-reaction of the antibody, and not to the autofluorescence. Overall, the combination of hypoxia labeling with Pimo-yne and CuAAC staining succeeded not only in the detection of hypoxia comparable to conventional pimonidazole IHC but also in decreasing background signals. The high hydrophilicity of the fluorophore and the linkers may contribute to the low non-specific binding to cellular proteins, resulting in low background signals.

## Conclusion

We developed Pimo-yne as a clickable hypoxia probe based on the pimonidazole skeleton. In cellular assay and tumor section imaging, Pimo-yne labeled hypoxia with high selectivity. Furthermore, the stained regions of the tissue sections were comparable to those of pimonidazole. These results indicate that incorporating a minimal alkyne tag into pimonidazole contributed to the similarity of hypoxia labeling property between Pimo-yne and pimonidazole. In this report, Cy5-PEG_4_-azide and CF647 azide were applied to hypoxia imaging of tumor tissue sections, which demonstrated high staining intensity and low background intensity. Due to the high signal-to-background ratio and the unnecessity of IHC, this system based on Pimo-yne labeling and CuAAC staining would be useful for an accurate and simple hypoxia detection method.

Pimo-yne developed in this study can label hypoxic regions similarly to pimonidazole and can be detected by various azide-tagged fluorescent dyes. In this study, it was demonstrated that a turn-on azide-tagged fluorescent dye, CF647 azide, can be applied to hypoxia imaging with Pimo-yne. The fluorescence of CF647 is “OFF” in the azide form, whereas it turns “ON” in the triazole form after the CuAAC reaction with Pimo-yne. Hence, CF647 may provide lower background signals, which could result in the detection of low signals of hypoxia undetectable thus far. The biological application of Pimo-yne is in progress in our laboratory.

### Supplementary Information

Below is the link to the electronic supplementary material.

Supplementary file1 (PDF 1091 kb)

## Data Availability

All data needed to evaluate the conclusions in the paper are present in the paper and/or the Supplementary Information.
