# Supplementary material for: Pimonidazole-alkyne conjugate for sensitive detection of hypoxia by Cu-catalyzed click reaction
Source: Anal Sci. 2024 Mar 13;40(6):1061–70. doi: 10.1007/s44211-024-00520-y (PMC11126502; doi:10.1007/s44211-024-00520-y)
Supplement: Supplementary file 1 — Supplementary file1 (PDF 1091 kb) [file 44211_2024_520_MOESM1_ESM.pdf]

Supplementary information

Pimonidazole-alkyne conjugate for sensitive detection of hypoxia by Cu-catalyzed click reaction

Iori Tamura[1], Daichi M. Sakamoto[1], Bo Yi[1], Yutaro Saito[1], Naoki Yamada[1 †], Yoichi Takakusagi[2,3], and Shinsuke Sando[1,4]*

[1]Department of Chemistry and Biotechnology, Graduate School of Engineering, The University of Tokyo, 7-3-1 Hongo, Bunkyo-ku, Tokyo, 113-8656, Japan.

[2]Quantum Hyperpolarized MRI Research Team, Institute for Quantum Life Science, National Institutes for Quantum Science and Technology, 4-9-1 Anagawa, Inage, Chiba-city, 263-8555, Japan.

[3]Institute for Quantum Medical Science, National Institutes for Quantum Science and Technology, 4-9-1 Anagawa, Inage, Chiba-city, 263-8555, Japan.

[4]Department of Bioengineering, Graduate School of Engineering, The University of Tokyo, 7-3-1 Hongo, Bunkyo-ku, Tokyo, 113-8656, Japan.

[†]Present address: Department of Physiology, National Defense Medical College, 3-2 Namiki, Tokorozawa, Saitama, 359-8513, Japan.

*Corresponding author
Shinsuke Sando
ssando@chembio.t.u-tokyo.ac.jp

Scheme S1 Synthesis of Cy5-PEG$_4$-azide

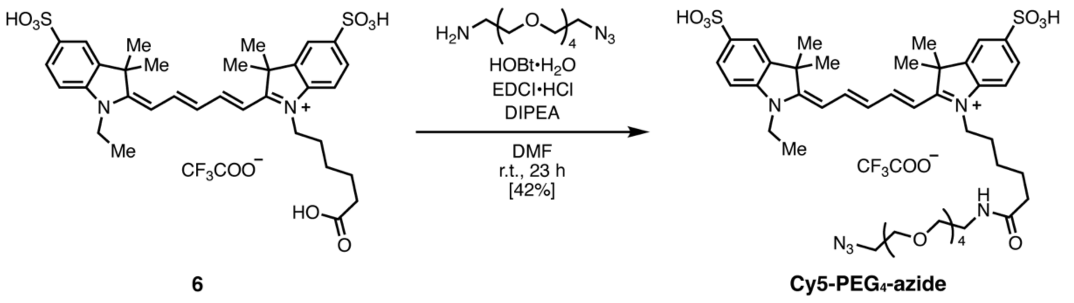

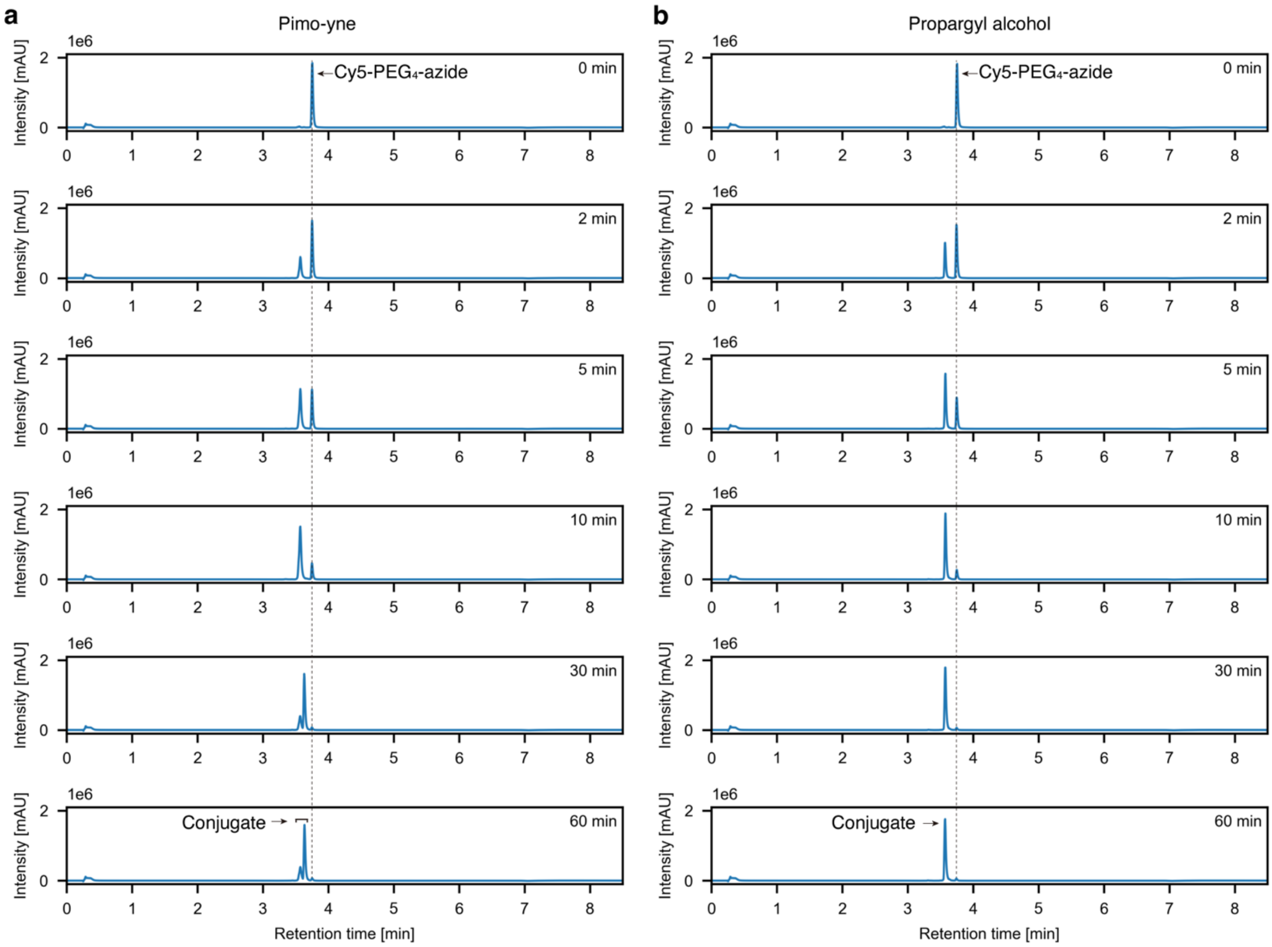

Fig. S1 UHPLC chromatograms for the time course of CuAAC reactions between Cy5-PEG$_4$-azide and **a** Pimo-yne and **b** propargyl alcohol. In **a**, part of pimonidazole-Cy5 conjugate was detected as reduced form. Products were monitored at 640 nm. The UHPLC was performed using two solvents (solvent A: H_2O containing 0.1% TFA; solvent B: acetonitrile containing 0.1% TFA). The column was first eluted with 10% solvent B for 1.5 min, followed by a linear gradient of 10–95% solvent B over 3 min

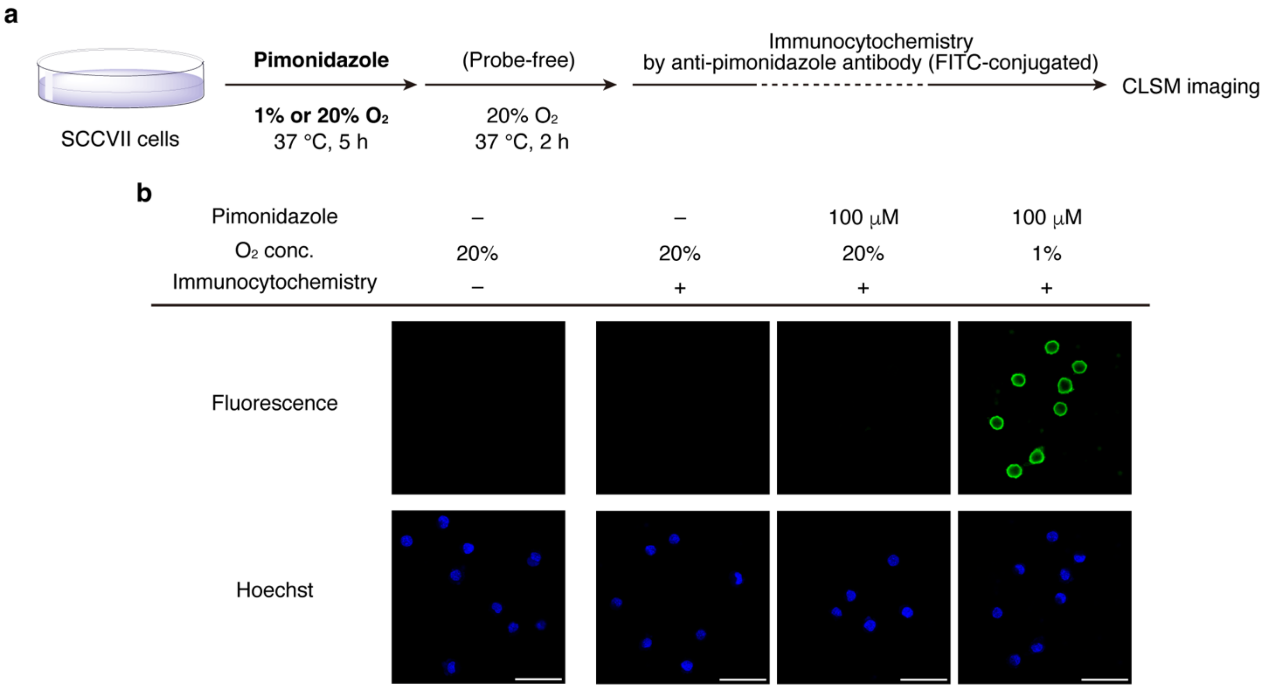

Fig. S2 Detection of hypoxic cells by pimonidazole. **a** Schematic illustration of the cellular assay. **b** CLSM imaging of cells treated with pimonidazole under hypoxia or normoxia, followed by immunocytochemistry staining. Scale = 50 μm

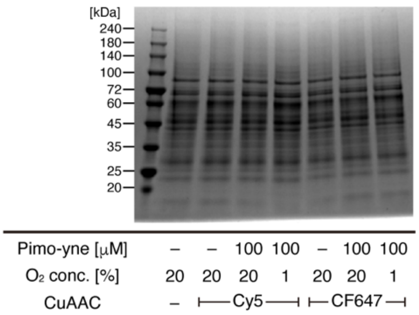

Fig. S3 CBB staining of the gel shown in Fig. 5c

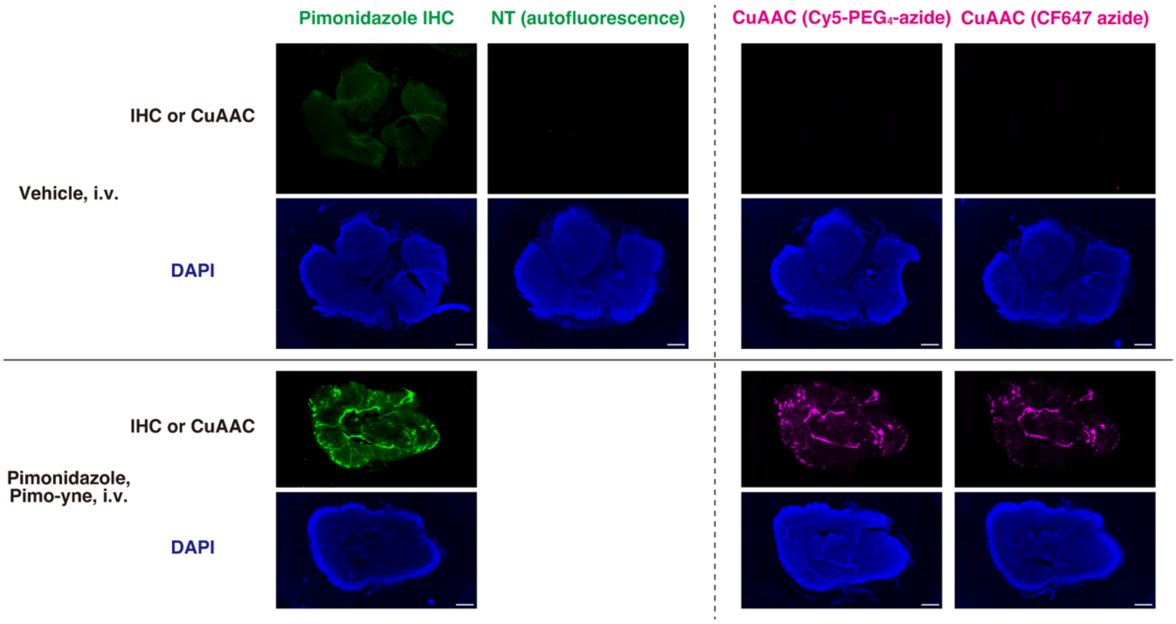

Fig. S4 The background signals of each staining on C26 tumor section (vehicle control). Upper: the IHC- or CuAAC-stained sections from a vehicle control mouse. NT indicates a non-treated section imaged under the same experimental and processing conditions as the IHC-stained section. Lower: the IHC- or CuAAC-stained sections from a pimonidazole/Pimo-yne administered mouse, which were imaged in the same experimental and processing conditions as the upper. DAPI: 4',6-diamidino-2-phenylindole. Magnification: ×4. Scale = 1 mm
